# Culling of Urban Norway Rats and Carriage of *Bartonella* spp. Bacteria, Vancouver, British Columbia, Canada

**DOI:** 10.3201/eid2808.211164

**Published:** 2022-08

**Authors:** Kaylee A. Byers, Michael J. Lee, Janet E. Hill, Champika Fernando, Laura Speerin, Christina M. Donovan, David M. Patrick, Chelsea G. Himsworth

**Affiliations:** The University of British Columbia School of Population and Public Health, Vancouver, British Columbia, Canada (K.A. Byers, M.J. Lee, C.M. Donovan, D.M. Patrick);; The University of British Columbia, Vancouver (K.A. Byers, C.G. Himsworth);; Canadian Wildlife Health Cooperative, Abbotsford, British Columbia, Canada (K.A. Byers, M.J. Lee, C.G. Himsworth);; University of Saskatchewan, Saskatoon, Saskatchewan, Canada (J.E. Hill, C. Fernando, L. Speerin);; British Columbia Centre for Disease Control, Vancouver (D.M. Patrick);; British Columbia Ministry of Agriculture, Abbotsford (C.G. Himsworth)

**Keywords:** *Bartonella*, bacteria, ecology, pest control, rats, *Siphonaptera*, zoonoses, vector-borne infections, Canada

## Abstract

We investigated the effects of culling on *Bartonella* spp. bacteria carriage among urban rats in Canada. We found that the odds of *Bartonella* spp. carriage increased across city blocks except those in which culling occurred. Removing rats may have prevented an increase in *Bartonella* spp. prevalence, potentially lowering human health risks.

Urban Norway rats (*Rattus norvegicus*) carry *Bartonella* spp., which are bacteria transmitted among rats and to humans through vectors including fleas (*1*). Infection in humans can result in fever, fatigue, myalgia, and endocarditis (*2*). In Vancouver, British Columbia, Canada, a serosurvey of residents of an underresourced neighborhood found that 3% of participants had been exposed to *B. tribocorum* (*3*), a species found in rats in this neighborhood (*4*), suggesting that rats may be an exposure source for humans in this area.

Although aimed at decreasing disease risks, culling methods (i.e., lethal removal) may increase zoonotic pathogen prevalence by altering normal behaviors that modify pathogen transmission (*5*,*6*). We sought to determine whether culling rats altered *Bartonella* spp. prevalence in rats and their fleas in the Downtown Eastside neighborhood of Vancouver. The University of British Columbia’s Animal Care Committee (A14-0265) approved study procedures.

## The Study

We trapped rats in 12 study sites (5 intervention, 7 control), each comprising 3 contiguous city blocks (36 total blocks) (Figure, panel A) during June 2016–January 2017 (Appendix). We placed 10 live traps (Tomahawk Live Traps, https://www.livetrap.com) in the alley of each block. We conducted the experiment in 3 trapping phases: before, during, and after the intervention (Figure, panel B). Before and after the intervention, we captured rats, gave each a numbered ear tag, and released it to its capture site. In the center block of intervention sites culling occurred during the second trapping phase. In flanking blocks (those adjacent to the intervention block) and control blocks, no culling occurred (Figure, panel A).

**Figure:**
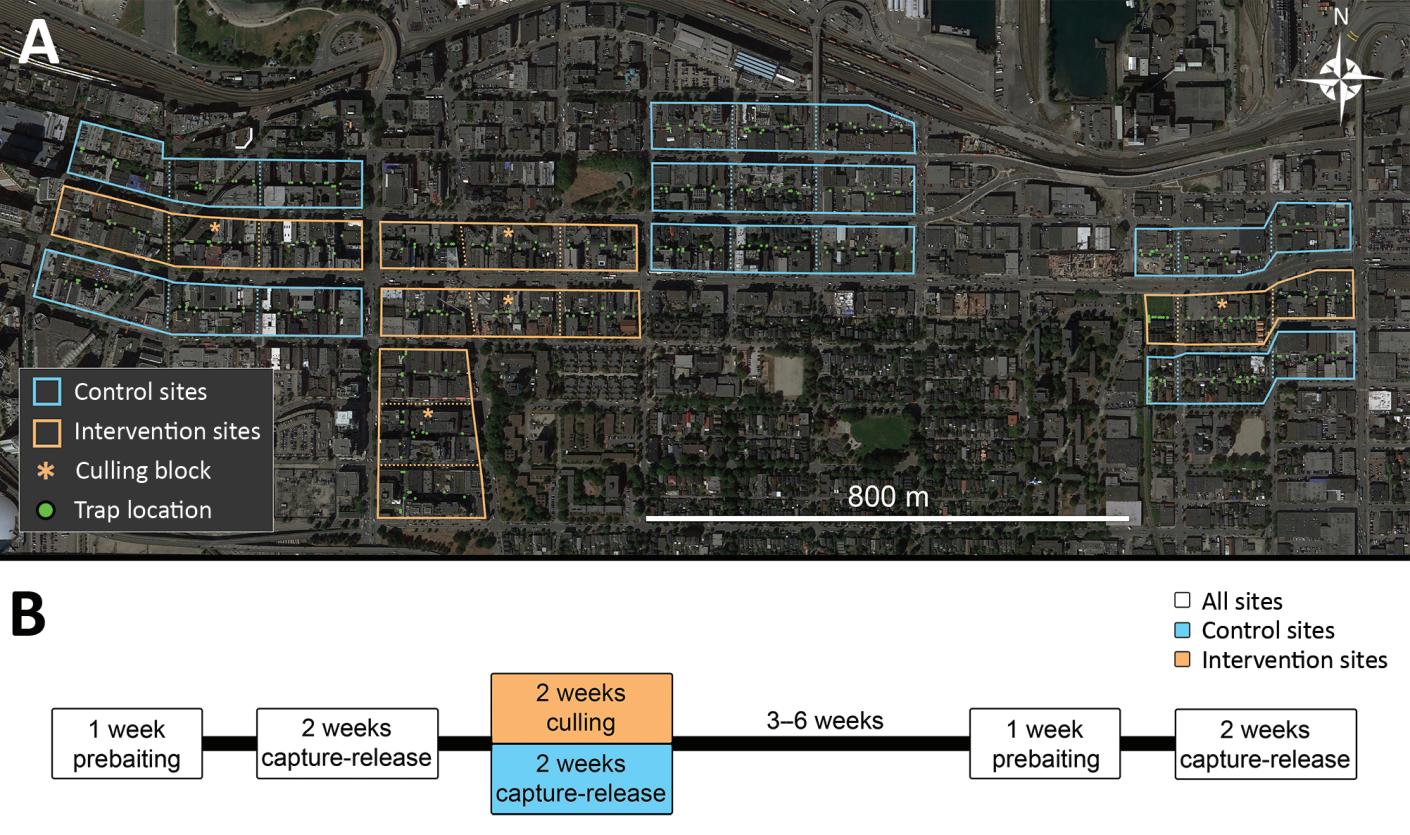
Trapping locations for Norway rats (*Rattus norvegicus*) caught in Vancouver, British Columbia, Canada. A) Trapping sites consisting of 3 contiguous city blocks. Each site was designated as a control or intervention site. Control sites did not involve culling (lethal animal removal); intervention sites included culling in the central block. B) Depiction of the study timeline. We first baited traps without capture to acclimatize rats to traps, then trapped and tagged rats with numbered ear tags and released the rats to their site of capture. After an intervention that involved culling rats in intervention sites, we resampled 3–6 weeks later to determine whether *Bartonella* spp. carriage differed between trapping periods before and after the intervention.

We collected blood from all rats via jugular puncture under isoflurane anesthesia. We collected fleas by brushing the coat. 

We identified fleas to species (*7*), and pooled <5 fleas per rat. We extracted DNA from rat blood and fleas using the DNEasy Blood and Tissue Kit (QIAGEN, https://www.qiagen.com). We tested DNA extracts for *Bartonella* spp. by real-time PCR. For rat blood, we used primers to detect a 380-bp segment of the citrate synthase gene (*gltA*) (*8*). For fleas, we used a probe-based real-time PCR assay to detect a 302-bp fragment of the *ssrA* gene (*9*). We conducted our analysis as described in Himsworth et al. (*10*).

We used generalized linear mixed models to assess the relationship between the intervention and *Bartonella* spp. carriage. We controlled for spatial clustering by city block as a random effect. We assessed positive or negative carriage by rats (model A) and fleas (model B) and the number of fleas per rat (model C). We analyzed carriage models A and B by logistic regression and model C by negative binomial regression. For all models, the intervention variable consisted of 4 categories indicating when rats or fleas were caught: before the intervention in all blocks; after the intervention in control blocks; after the intervention in flanking blocks; and after the intervention in intervention blocks.

We used a hypothesis-testing model building approach to estimate the effect of the intervention while accounting for covariates (Table). We retained covariates if they confounded the relationship between the intervention and the outcome (i.e., if they changed the effect of any level of the intervention by >10% or if their association with the outcome and intervention had p<0.25). We also kept independent predictors of the outcome if they significantly improved the model, as indicated by a likelihood ratio test result of p<0.05; that test compared 2 nested models, each with the intervention variable and all confounders present, but with and without the potential predictor variable.

**Table:** Mixed effects logistic regression models of the effect of intervention on *Bartonella* spp. carriage by Norway rats (*Rattus norvegicus*), Vancouver, British Columbia, Canada*

Variable	*Bartonella* prevalence, no. positive/no. tested (%)	Bivariable models				Final model†	
		Unadjusted OR (95% CI)	p value in model	LRT p value‡		Adjusted OR (95% CI)	p value in model
Intervention							
Rats caught before the intervention in all blocks	58/267 (22)	Referent	Referent	Referent		Referent	Referent
Rats caught after the intervention in control blocks	24/109 (22)	1.26 (0.67–2.39)	0.47	<0.01		2.68 (1.22–6.67)	0.02
Rats caught after the intervention in flanking blocks	6/37 (16)	0.56 (0.18–1.46)	0.26	NA		7.26 (1.56–38.17)	0.01
Rats caught after the intervention in intervention blocks	2/41 (5)	0.12 (0.02–0.46)	<0.01	NA		2.03 (0.22–15.41)	0.50
Sex							
F	38/221 (17)	Referent	Referent	Referent		NA	NA
M	52/233 (22)	1.32 (0.82–2.14)	0.26	0.26		NA	NA
Sexual maturity							
Juvenile	34/177 (19)	Referent	Referent	Referent		NA	NA
Mature	56/277 (20)	0.98 (0.60–1.63)	0.95	0.95		NA	NA
Wound presence							
Absent	59/339 (17)	Referent	Referent	Referent		Referent	Referent
Present	31/115 (27)	1.67 (0.97–2.81)	0.06	0.06		1.49 (0.83–2.63)	0.17
Weight§	NA	1.04 (0.81–1.32)	0.75	0.75		NA	NA
Presence of fleas on rats							
Absent	46/261 (18)	Referent	Referent	Referent		NA	NA
Present	44/193 (23)	1.39 (0.86–2.25)	0.18	0.18		NA	NA
No. fleas on rat	NA	1.02 (0.95–1.09)	0.50	0.52		NA	NA
Flea index#	NA	1.13 (0.90–1.43)	0.31	0.32		NA	NA
Presence of positive fleas per rat							
Absent	67/376 (18)	Referent	Referent	Referent		Referent	Referent
Present	23/78 (30)	1.83 (1.00–3.25)	0.04	0.05		1.94 (1.00–3.69)	0.05
Season							
Summer, June–August	16/124 (13)	Referent	Referent	Referent		Referent	Referent
Fall, September–November	65/208 (31)	3.16 (1.59–6.73)	<0.01	<0.01		2.90 (1.32–6.31)	<0.01
Winter, December–March	9/122 (7)	0.50 (0.18–1.30)	0.15	NA		0.16 (0.03–0.68)	0.02

*OR refers to the odds of *Bartonella* spp. carriage among rats in each group relative to the reference group for that variable. Variables were included in the final model if they confounded the relationship between the intervention; and the outcome (changed the effect of any level of the intervention by >10% and/or were associated with the outcome and intervention, p<0.25) or if they were independent predictors that improved the model as indicated by a significant (p<0.05 likelihood ratio test with all confounders and intervention present). LRT, likelihood ratio test; NA, not applicable; OR, odds ratio. †Final multivariable model: *Bartonella* status ~ intervention + wound presence + presence of positive fleas per rat + season + (city.block). ‡Likelihood ratio test comparing the generalized linear mixed model with and without the indicated variable; p<0.05 indicates that the variable significantly improved the model with all confounders and as such was a significant predictor and was retained in the final model. §Scaled and centered around its mean. #Average number of fleas per rat per city block.

We trapped 512 Norway rats; 206 (40.2%) of them had fleas. The median number of fleas per rat was 0 (range 0–58; mean 1.18). All fleas were *Nosopsyllus fasciatus.* We obtained blood from 454 rats; 90 (20%) tested positive for *Bartonella* spp. We tested 201 flea pools; 86 (42.8%) tested positive for *Bartonella* spp. (Table). In the final model A, which contained the variables season, presence of *Bartonella* spp.–positive fleas, and wound presence as covariates, the odds of *Bartonella* spp. carriage were significantly higher among rats caught after the intervention in control blocks (odds ratio [OR] 2.68; 95% CI 1.22–6.67) and flanking blocks (OR 7.26; 95% CI 1.56–38.17), but not in the intervention blocks (OR 2.03; 95% CI 0.22–15.41), when compared with the odds of carriage before the intervention in all block types (Table). We saw no association between the intervention and the number of fleas per rat or *Bartonella* spp. carriage by fleas.

## Conclusions

The prevalence of *Bartonella* spp. bacteria among rats in this neighborhood has been shown to increase in the fall (*4*). Our study suggests that culling rats may have prevented this increase within the blocks where culling occurred.

Removing rats may change how individual rats interact within colonies, which alters pathogen transmission. *Bartonella* spp. transmission via fleas (*1*) requires close contact among individual rats. Rats burrow communally, establishing a network of chambers with some shared nests (*11*). Those nests promote close contact among rats and act as a source of fleas that spend time in the nest (*12*). Decreased rat population density may lessen nest sharing and behaviors such as social grooming, thereby reducing opportunities for fleas to transmit *Bartonella* spp. among individual rats. A reduction in *Bartonella* spp. prevalence may decrease exposure risk for humans, but the relationship between rodents, vectors, pathogens, and humans is complex (*13*). For example, although a previous study revealed that residents in this neighborhood had been exposed to *Bartonella* spp. (*3*), it is unclear whether this exposure was associated with rats and to what extent humans encounter fleas. Furthermore, for other fleaborne pathogens such as *Yersinia pestis* (agent of the plague), culling rats may increase disease transmission to humans as fleas seek new hosts (*14*). Understanding how rat abundance and rat removal impacts intraspecies and interspecies dynamics and pathogen prevalence is necessary to anticipate management impacts on pathogen transmission.

Whereas our intervention involved removing rats and their fleas, we did not observe a change in the number of fleas on rats. The steady number suggests that culling did not reduce flea abundance, perhaps because *N. fasciatus* fleas also reside in the burrows, such that the number of fleas per rat does not reflect the total number of fleas in a city block (*12*). It is possible that our intervention removed a negligible proportion of the flea population. In addition, we did not observe a change in the odds of *Bartonella* spp. carriage among fleas. A past study in this neighborhood showed that *Bartonella* spp. carriage among rats was not related to flea presence or abundance; therefore, the role of *N. fasciatus* fleas in the ecology of *Bartonella* spp. in this ecosystem remains enigmatic (*15*).

Our findings counter a study of *Leptospira interrogans* using the same experimental design, in which culling was associated with an increased odds of infection among rats (*5*). This difference is likely attributable to differences in transmission; *L. interrogans* is spread via urine (*13*) and *Bartonella* spp. via fleas (*1*). Culling may alter a variety of social interactions (e.g., fighting, nest-sharing, grooming) which affect the spread of these pathogens differently. Together, these studies illustrate the complexity of managing rat-associated zoonoses; the intervention may have opposite effects on different pathogens. Indeed, past literature has shown that culling wildlife to control zoonoses can have unpredictable consequences (*6*) and that ecosystem-based approaches that manage the human–wildlife interface may be more effective.

Appendix Additional information about the culling of urban Norway rats and association with carriage of *Bartonella* spp. bacteria, Vancouver, British Columbia, Canada.
