# Supplementary material for: Culling of Urban Norway Rats and Carriage of Bartonella spp. Bacteria, Vancouver, British Columbia, Canada
Source: Emerg Infect Dis. 2022 Aug;28(8):1659–63. doi: 10.3201/eid2808.211164 (PMC9328906; doi:10.3201/eid2808.211164)
Supplement: Appendix — Additional information about the culling of urban Norway rats and association with carriage of Bartonella spp. bacteria, Vancouver, British Columbia, Canada. [file 21-1164-Techapp-s1.pdf]

Article DOI: https://doi.org/10.3201/eid2808.211164

Culling of Urban Norway Rats and Carriage of *Bartonella* spp. Bacteria, Vancouver, British Columbia, Canada

Appendix

Methodology

Trapping

Trapping was conducted in the Downtown Eastside neighborhood of Vancouver, Canada from June 2016–January 2017. The study included 12 study sites, each consisting of 3 city blocks (total = 36); 5 were designated as intervention sites and 7 were designated as controls. In intervention sites, lethal trapping occurred in the central block, while the 2 adjacent blocks were considered non-lethal flanking blocks. This designation was meant to account for potential rat movement between contiguous alleyways that might occur in response to the intervention. In control sites, no lethal trapping occurred.

To capture rats, we placed 10 Tomahawk rigid traps (Tomahawk Live Traps, Hazelhurst, WI, USA) in the alley of each city block. Traps were covered with stainless steel covers (Integrated Pest Supplies Ltd, New Westminster, BC, Canada) to prevent vandalism and to minimize contact between captured rats and humans. Traps were chained to immovable objects in alleyways to prevent removal.

Three study sites were trapped at a time, including a total of 9 city blocks and 90 active traps. Prior to active trapping, we prebaited traps for 1 week by baiting and fixing traps open to acclimatize rats to traps and to increase the likelihood of rats entering traps during the active trapping period (*1*,*2*). Traps were baited with a peanut butter–oat mixture and Hydrogel (ClearH2O, Westbrook, ME, USA) to provide a water source.

During active trapping periods, traps were set at 4 PM and checked every morning before 7 AM. Traps were active 5 days a week and on the sixth and seventh days, traps were fixed open

and baited to reacclimatize rats to traps (*2*). To minimize the potential of pathogen spread among rats due to contamination, all traps were sanitized in 10% bleach after coming into contact with any rat and after any prebaiting period (*3*).

Active trapping occurred for a total of 6 weeks, divided into 2-week trapping periods. These trapping periods were designated as before; during; or after the intervention. During the before and after periods, all rats that were caught were released at their site of capture after sampling (detailed below). During the intervention period, rats caught in intervention blocks were euthanized by intracardiac injection with pentobarbital after sampling.

Rat Sampling

Trapped rats were transported to a mobile laboratory-van where they were covered with a blanket to minimize stress before sampling. Each rat was transferred into an inhalation induction chamber (Kent Scientific, Torrington, CT, USA) and anesthetized with 5% isoflurane in oxygen using an isoflurane vaporizer (Associated Respiratory Veterinary Services, Lacombe, AB, Canada). Anesthesia was maintained throughout sampling through a nose cone administering the anesthetic.

To identify rats which had been previously caught, each rat was given a unique laser-etched ear-tag (Kent Scientific). The following demographic characteristics were recorded for each rat: bodyweight (grams); sexual maturity (maturity was determined as males with scrotal testes and females with a perforate vagina); sex (male or female); and the presence/absence of bite wounds. Bite wounds were considered an important characteristic in this study because they might indicate close contact among individuals (which would allow for fleas to spread among rats) and because *Bartonella* spp. is spread in flea feces and the bacteria is introduced through openings in the skin (*4*).

Blood was collected from each rat via the jugular and then stored in heparin-coated microtainers (BC, Mississauga, Canada). Fleas were collected from rats in 2 ways. First, the majority of fleas vacated the rat while in the induction chamber and were collected from the induction chamber using tweezers. Second, the coat of each rat was brushed thoroughly over a collection bowl to dislodge any remaining fleas.

Prior to release at their location of capture, rats were allowed to fully recover from the anesthesia (≈15–30 minutes). Individuals that were recaptured were resampled if they were

caught more than 7 days after their prior sampling. This period was informed by guidelines from the University of British Columbia's Animal Care Committee to ensure that rats had sufficient time to recover between blood collection periods.

DNA Extraction

Rat blood and fleas were stored at −80°C before DNA extraction. Fleas were identified to species by observing them under a compound microscope at 40× (*5*). To extract DNA from fleas, we pooled up to 5 fleas per rat. Flea pools were surface-sterilized in 10% bleach and then rinsed in nuclease-free water and twice in 100% ethanol to remove bacteria from the external body. Flea pools were then crushed using a sterile scalpel. DNA from rat blood and fleas was extracted using the QIAgen DNEasy Blood and Tissues Kit following the manufacturer's protocol.

Detailed methods for *Bartonella* spp. testing are outlined in Himsworth et al. (*6*).

Statistical Analysis

Model A The impact of the intervention on *Bartonella* spp. carriage among rats.

To assess the impact of the intervention on *Bartonella* spp. carriage among rats we built mixed effects logistic regression models, otherwise known as generalized linear mixed models, or GLMMs. The outcome for Model A was the *Bartonella* spp. status of individual rats (positive or negative). The intervention was expressed statistically as a categorical variable consisting of four levels and was an indicator of whether rats were captured: 1) in the 2 weeks before the intervention in any block type (the reference category); 2) in the 2-week period after the intervention in control blocks; 3) in the 2-week period after the intervention in flanking blocks; or 4) in the 2-week period after the intervention in intervention blocks. Although we did not perform the intervention in control and flanking blocks, we considered the 2-week trapping periods after the intervention in control and flanking blocks independently to detect temporal changes in prevalence not related to the intervention. Finally, we included an indicator of the city-block as a random effect in all steps of the modeling process to control for heterogenous prevalence of *Bartonella* spp. among blocks (*7*).

For each GLMM we took a hypothesis testing model building approach to estimate the effect of the intervention on the *Bartonella* spp. status of each rat while controlling for important covariates. Potential covariates included sex (male or female), sexual maturity (juvenile or mature), weight (g), presence of fleas (present or absent), presence of bite wounds (present or

absent), number of fleas (count), flea index (average number of fleas per rat in a city-block), presence of positive fleas (present or absent), and season (summer, fall, winter). Summer was designated as June–August, fall was September–November, winter was December–March.

We included covariates in the model that were either confounders of the relationship between the intervention variable and the outcome or that were independent significant predictors of the outcome. In step one, we assessed whether covariates were confounders. To do this, we first examined the relationships between each potential confounder and the outcome in separate GLMMs containing only that variable and the random effect of the city block. Variables associated with the outcome ($p \leq 0.25$) were then assessed for their impact on the intervention variable. To do this, we evaluated whether the inclusion of each covariate, that was associated with the outcome ($p \leq 0.25$), in a GLMM containing that variable and the intervention variable, changed the effect estimate of any level of the intervention (i.e., rats caught before the intervention in any block type, rats caught after the intervention in control blocks, etc.) by ≥10% relative to its effect estimate in a GLMM containing only the intervention variable. If the variable met both of these criteria, they were considered confounders. In step two, variables that did not meet the confounder criteria, but were still significantly ($p \leq 0.05$) associated with the outcome were considered as independent significant predictors. Each of these variables were entered into the model containing the intervention and identified confounders. Variables were retained in the model if they substantially decreased the standard error of any level of the intervention variable effect estimates and/or if they significantly improved the model as evidenced by a significant likelihood ratio test ($p \leq 0.05$) that compared the overall model with and without that variable. The final model contained all confounders and significant predictors that met these criteria.

Thirty-three animals were recaptured; 13 were recaptured in the 2 weeks before the intervention and 20 were recaptured after. For rats that were recaptured within the same period (before or after) rat characteristics were either averaged (e.g., average weight) or for characteristics that were categories (i.e., juvenile vs. mature) the value for the latest capture was used. Further, if any of these recaptured rats tested positive once, then they were considered to be *Bartonella* spp. positive. The resulting dataset included 454 rats.

Model B The impact of the intervention on Bartonella spp. carriage among fleas.

The impact of the intervention on *Bartonella* spp. carriage by fleas was modeled following the same GLMM building process as for rats. However, in this model the outcome was the *Bartonella* spp. status of flea pools (positive or negative), and the intervention variable categorized whether fleas were taken from rats before the intervention in any block type, after the intervention in control blocks, after the intervention in flanking blocks, or after the intervention in intervention blocks.

Among 33 recaptured rats, 7 had fleas that were tested. If these rats were captured in the same 2-week trapping period, the average number of fleas collected across the captures was used and the fleas were considered positive from that rat if they tested positive in $\geq$1 recapture in that 2-week period. The dataset included 201 rats from which fleas were collected.

Model C The impact of the intervention on the number of fleas on rats.

To assess whether the intervention influenced the number of fleas counted on each rat, we used a negative binomial GLMM to account for overdispersion in the outcome. The outcome was the number of fleas counted on each rat. This model was built following the same procedure as for Models A and B. This analysis used the same 454 rats as Model A.

References:

1. Barnett SA. The rat: a study in behaviour. Chicago, IL: Aldine Publishing Co. 1963.

2. Byers KA, Lee MJ, Bidulka JJ, Patrick DM, Himsworth CG. Rat in a cage: trappability of urban Norway rats (*Rattus norvegicus*). Front Ecol Evol. 2019;7:68. https://doi.org/10.3389/fevo.2019.00068

3. Health Canada, Public Health Agency of Canada. Pathogen safety data sheets: infectious substances – *Leptospira interrogans*. P. 1–3. 2011 [cited 2022 Feb 6]. http://www.phac-aspc.gc.ca/lab-bio/res/psds-ftss/msds95e-eng.php

4. Gutiérrez R, Krasnov B, Morick D, Gottlieb Y, Khokhlova IS, Harrus S. *Bartonella* infection in rodents and their flea ectoparasites: an overview. Vector Borne Zoonotic Dis. 2015;15:27–39. PubMed https://doi.org/10.1089/vbz.2014.1606

5. Holland GP. The fleas of Canada, Alaska, and Greenland (Siphonaptera). Mem Entomol Soc Can. 1985;117(S130):3–632. https://doi.org/10.4039/entm117130fv

6. Himsworth CG, Byers KA, Fernando C, Speerin L, Lee MJ, Hill JE. When the sum of the parts tells you more than the whole: the advantage of using metagenomics to characterize *Bartonella* spp. infections in Norway rats (*Rattus norvegicus*) and their fleas. Front Vet Sci. 2020;7:584724. PubMed https://doi.org/10.3389/fvets.2020.584724

7. Himsworth CG, Bai Y, Kosoy MY, Wood H, DiBernardo A, Lindsay R, et al. An investigation of *Bartonella* spp., *Rickettsia typhi*, and Seoul hantavirus in rats (*Rattus* spp.) from an inner-city neighborhood of Vancouver, Canada: is pathogen presence a reflection of global and local rat population structure? Vector Borne Zoonotic Dis. 2015;15:21–6. PubMed https://doi.org/10.1089/vbz.2014.1657
